# Mutations in haemagglutinin that affect receptor binding and pH stability increase replication of a PR8 influenza virus with H5 HA in the upper respiratory tract of ferrets and may contribute to transmissibility

**DOI:** 10.1099/vir.0.050526-0

**Published:** 2013-06

**Authors:** Holly Shelton, Kim L. Roberts, Eleonora Molesti, Nigel Temperton, Wendy S. Barclay

**Affiliations:** 1 Division of Infectious Diseases, Imperial College London, St Mary’s Campus, London, UK; 2 Viral Pseudotype Unit, School of Pharmacy, University of Kent, Anson Building, Chatham Maritime ME4 4TB, UK

## Abstract

The H5N1 influenza A viruses have circulated widely in the avian population for 10 years with only sporadic infection of humans observed and no sustained human to human transmission. Vaccination against potential pandemic strains is one strategy in planning for future influenza pandemics; however, the success of live attenuated vaccines for H5N1 has been limited, due to poor replication in the human upper respiratory tract (URT). Mutations that increase the ability of H5N1 viruses to replicate in the URT will aid immunogenicity of these vaccines and provide information about humanizing adaptations in H5N1 strains that may signal transmissibility. As well as mediating receptor interactions, the haemagglutinin (HA) protein of influenza facilitates fusion of the viral membrane and genome entry into the host cell; this process is pH dependent. We have shown in this study that the pH at which a panel of avian influenza HA proteins, including H5, mediate fusion is higher than that for human influenza HA proteins, and that mutations in the H5 HA can reduce the pH of fusion. Coupled with receptor switching mutations, increasing the pH stability of the H5 HA resulted in increased viral shedding of H5N1 from the nasal cavity of ferrets and contact transmission to a co-housed animal. Ferret serum antibodies induced by infection with any of the mutated H5 HA viruses neutralized HA pseudotyped lentiviruses bearing homologous or heterologous H5 HAs, suggesting that this strategy to increase nasal replication of a vaccine virus would not compromise vaccine efficacy.

## Introduction

Highly pathogenic avian influenza (HPAI) virus H5N1 has circulated widely in a diverse range of avian species for nearly a decade. It has also caused disease in several mammals, including humans, often with lethal consequence. The current human mortality rate for infection with HPAI H5N1 is ~58 % in WHO confirmed cases (WHO, 2012b). To date no sustained human to human transmission of the virus has been observed, with most human cases resulting from direct contact with sick birds (Aditama *et al.*, 2012; Ungchusak *et al.*, 2005; Wang *et al.*, 2008). However, the concern remains that, if human transmissibility was acquired, a severe pandemic could result.

Vaccination is the most effective method for controlling a newly emerged influenza virus in the community. Inactivated influenza vaccines for a newly emerged subtype such as H5 to which the human population is not primed may require high antigen doses, two or more immunizations, and/or adjuvants (Couch *et al.*, 1997; Subbarao *et al.*, 2006). Live attenuated vaccines (LAIVs) would allow greater vaccine coverage because more doses can be generated from each infected egg. However, efforts to trial LAIVs for H5 in humans have been largely unsuccessful so far, with poor antibody responses (Karron *et al.*, 2009). This can be explained by the cold-adapted (*ca*) genetic backbone of the LAIV driving virus replication to the cooler temperatures of the upper respiratory tract (URT), but the avian-adapted H5 haemagglutinin (HA) protein having low affinity for the α2,6 linked sialic acid (SA) receptors that predominate there.

Indeed, a prerequisite for an avian influenza virus to acquire transmissibility between mammals is the switch in SA linkage preference of HA from α2,3 linked SA to α2,6 linked SA, which will allow greater replication in the URT, the site of transmission. We and others have described mutations that might mediate this switch (Auewarakul *et al.*, 2007; Ayora-Talavera *et al.*, 2009; Chutinimitkul *et al.*, 2010; Gambaryan *et al.*, 2006; Gao *et al.*, 2009; Harvey *et al.*, 2004; Maines *et al.*, 2011; Su *et al.*, 2008; Watanabe *et al.*, 2011; Yamada *et al.*, 2006). Several groups have engineered recombinant H5N1 viruses that contain α2,6 SA-adapting mutations in the HA and tested transmissibility in ferrets, the best experimental animal model to indicate the potential for human influenza transmission (Chen *et al.*, 2012a; Harvey *et al.*, 2004; Maines *et al.*, 2011). However, receptor binding switches alone may not mediate a complete switch of host range for H5N1 viruses. Indeed, Maines *et al.* (2011) showed that receptor switching mutations at the receptor binding site, Q226L and G228S, in H5 HA resulted in lower viral shedding from inoculated ferrets, and transmission remained as inefficient as for wild-type H5N1 viruses (Maines *et al.*, 2011). However, Chen *et al.* (2012a) showed that insertion of three receptor binding mutations 196R/226L/228S into a clade 2.2 H5N1 virus combined with replacement of the avian virus NA (neuraminidase) gene by a human H3N2 virus NA resulted in some respiratory droplet transmission in this model. Very recently two reports of mammalian transmissible H5N1 viruses indicated a requirement for receptor binding changes in the H5 HA to increase the α2,6 SA affinity in combination with other changes in the HA protein (Herfst *et al.*, 2012; Imai *et al.*, 2012).

Wang *et al.* (2010) investigated whether receptor binding mutations in HA can be used to increase the nasal replication of H5 LAIV. They introduced the Q226L and G228S mutations in combination with loss of the glycosylation site at N158 and showed increased replication in ferrets. However, protection mediated by this LAIV was not cross-protective for viruses that retained the HA glycosylation site at residue 158.

One property that has been somewhat overlooked until now, which may affect the ability of avian influenza viruses to replicate in the mammalian respiratory tract, is the pH stability of the HA protein. HA is the fusogenic protein of the virus. After entry of the virus particle to the host cell, in the acidified environment of the endosome, HA undergoes an irreversible conformational change that exposes the hydrophobic fusion peptide and initiates membrane fusion leading to release of the viral genome into the cytoplasm (Skehel & Wiley, 2000). The pH at which HA undergoes this transformation varies between different strains of the virus (Beyer *et al.*, 1986). During adaptation to growth in cell culture or to different host species, the pH at which HA fusion occurs is altered (Giannecchini *et al.*, 2006; Lin *et al.*, 1997). Viruses bearing HA protein that fuses at a high pH release their genomes more rapidly upon cell entry and this can bestow a replicative advantage under certain conditions. However, viruses with HAs that require a lower pH to fuse display increased environmental stability and may transmit more efficiently as a result (Reed *et al.*, 2010). The way this balance plays out for any individual virus is likely influenced by the environmental factors encountered during transmission events. For example, transmission in overcrowded poultry houses or by virus shedding into brackish water by ducks may select for HA proteins with a high pH of fusion, whereas transmission through the air into the relatively acidic environment of the human nasal cavity (England *et al.*, 1999; Washington *et al.*, 2000) might require a more pH-stable HA. Indeed, the ability of H5N1 viruses to replicate in the mouse URT was shown by Krenn *et al.* (2011) to be enhanced by mutations that stabilized the HA. We therefore speculated that mutations that lowered the pH of fusion of H5 HA might enhance its replication in the upper airways of ferrets, inducing a more robust immune response and concomitantly lead to a transmissible phenotype, at least in combination with other HA changes that affect receptor specificity and NA changes that affect virus release.

## Results

### Human-adapted HA proteins fuse at lower pH than avian virus HAs

**Fig. 1.**
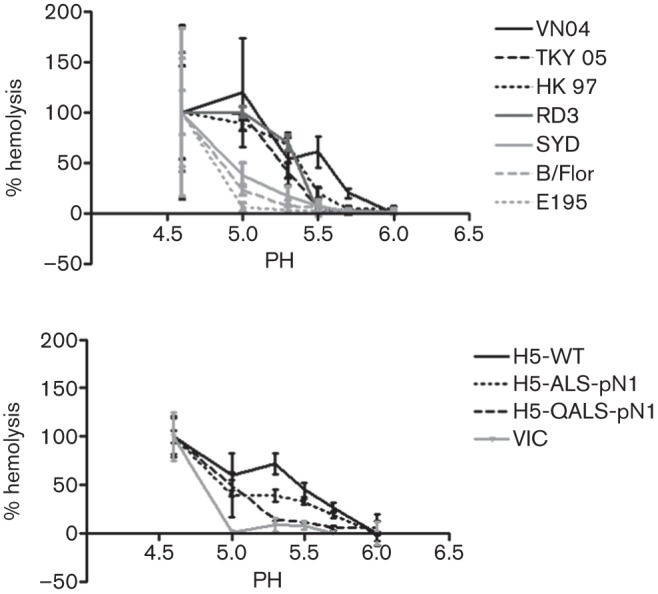
Influenza viruses with human- or avian-adapted HA were tested for their ability to cause fusion in hRBCs at varying pHs. A total of 64 HA units of each virus was mixed with hRBCs in differing pH conditions and the degree of fusion monitored by the release of haemoglobin by measuring the optical density at 405 nm. Each virus was tested in quadruplicate and the percentage of maximum fusion achieved at each pH was calculated for each virus. The means of quadruplicate tests were plotted with sds indicated. (a) Comparison between human-adapted influenza strains E195 - A/England/195/2009 (pH 1N1), B/Flor - B/Florida/4/2006 and SYD - A/Sydney/5/1997 (H3N2) (grey lines), and viruses bearing the surface antigens of HPAI avian viruses from which the multi-basic site had been removed from the HA on an internal genetic background of A/PR/8/34 virus as follows: VN04 - A/Vietnam/1194/04 (H5N1), TKY05 - A/turkey/Turkey/01/05 (H5N1), HK97 - A/Hong/Kong/156/97 (H5N1) and RD3 - A/chicken/Italy/13474/99 (H7N1) (black lines). (b) H5 HA mutant viruses with the multi-basic site engineered away from H5 HA and on an internal genetic background of A/PR/8/34 (H5-ALS-pN1 and H5-QALS-pN1) (black lines) were compared to H5-wt (black line) and a human virus (VIC - A/Victoria/3/75 (H3N2) (grey line). Table 1 shows the details of the genetic composition of each virus.

We assessed a panel of viruses possessing the HA and NA genes of either human transmissible viruses or avian influenza isolates (see Table 1) for the pH at which the HAs triggered membrane fusion. This was achieved by mixing 64 HA units of each virus with human red blood cells (hRBCs) at 4 °C. After binding between HA and SA on the erythrocytes had taken place, the mix was exposed to gradually decreasing pH at 37 °C. Fusion of membranes triggered by the HA conformational change was measured by the release of haemoglobin from the erythrocytes. The human transmissible virus HAs required a lower pH (<pH 5.3) to undergo fusion in comparison to the avian virus HAs (<pH 5.5) (Fig. 1a).

**Table 1.** Genetic composition of viruses used in the study

Virus name	H5 HA gene mutation (A/Vietnam/1194/2004)	NA gene	Phenotype
H5-wt*	Wild-type	A/Vietnam/1194/2004	Wild-type
H5-LS*	Q226L/G228S	A/Vietnam/1194/2004	↑α2,6 SA binding
H5-ALS*	T160A/Q226L/G228S	A/Vietnam/1194/2004	↑α2,6 SA binding
H5-ALS-pN1*	T160A/Q226L/G228S	A/England/195/2009	↑α2,6 SA binding+pandemic H1N1 NA
H5-QALS-pN1*	H24Q/T160A/Q226L/G228S	A/England/195/2009	↑α2,6 SA binding+↓pH of HA fusion+pandemic H1N1 NA

Virus name	HA gene	NA gene	Phenotype
VN04 (H5 wt)*	A/Vietnam/1194/2004	A/Vietnam/1194/2004	Avian H5N1 (minus MBS)
HK97*	A/Hong Kong/156/97	A/Hong Kong/156/97	Avian H5N1 (minus MBS)
TKY05*	A/turkey/Turkey/01/2005	A/turkey/Turkey/01/2005	Avian H5N1 (minus MBS)
RD3*	A/chicken/Italy/13474/99	A/chicken/Italy/13474/99	Avian H7N1 (minus MBS)
**SYD**	A/Sydney/2007/97	A/Sydney/2007/97	Human H3N2
**B/Flor**	B/Florida/04/2006	B/Florida/04/2006	Human influenza B
**VIC**	A/Victoria/3/75	A/Victoria/3/75	Human H3N2
**E195**	A/England/195/2009	A/England/195/2009	Human pH 1N1

* Viruses contain the six internal gene segments from A/PR/8/34.

### A single point mutation lowers the pH of fusion of H5 HA

We generated a panel of recombinant influenza viruses possessing the H5 HA (A/Vietnam/1194/2004) into which well characterized humanizing receptor binding site mutations were engineered, Q226L and G228S alone or coupled with the mutation T160A, which abrogates glycosylation at residue 158 (Chutinimitkul *et al.*, 2010; Gao *et al.*, 2009; Wang *et al.*, 2010). We then additionally engineered another HA mutation, H24Q, which has been documented to lower the pH of fusion (Reed *et al.*, 2009, 2010). We paired these mutated H5 HAs with either the matched A/Vietnam/1194/2004 N1 NA gene or the N1 NA gene from a prototypic isolate of the 2009 H1N1 human pandemic virus (A/England/195/2009) since this NA clearly supports efficient transmission in humans and could be acquired during a reassortment with H5N1 viruses (see Table 1). For all of the H5 recombinant viruses we generated, we removed the multi-basic cleavage site [a known major virulence factor (Hatta *et al.*, 2001)] from the HA gene, and combined the H5 HA and N1 NA genes with the remaining six genome segments from the high growth reassortant vaccine donor strain of influenza virus, A/PR/8/34. Thus, our H5 recombinant viruses were similar in genetic makeup to the reverse genetics (RG) vaccines that have been used widely to generate and test pre-pandemic H5 inactivated vaccines (WHO, 2012a). Such viruses have been extensively tested in chickens, mice and ferrets, and demonstrated to lack the highly pathogenic phenotype [reviewed by Horimoto & Kawaoka (2009)].

We tested the effect of the engineered HA mutation (H24Q) on the pH of fusion. The human H3N2 virus (A/Victoria/3/75) did not undergo efficient fusion until exposed to a pH of <5.0 in line with the other human viruses tested in Fig. 1(a). In contrast, membrane fusion of the H5-wt virus was triggered at a pH of 5.7. The threshold pH at which fusion first occurred was not affected by the receptor specificity of the HA or by switching the avian-adapted N1 NA for the NA from the 2009 pH 1N1 virus (compare H5-wt virus with H5-ALS-pN1 in Fig. 1b). The single amino acid change H24Q lowered the H5 HA pH of fusion, such that fusion was not detected at a pH of 5.3 or above.

### pH of fusion does not affect multicycle replication in MDCK cells

**Fig. 2.**
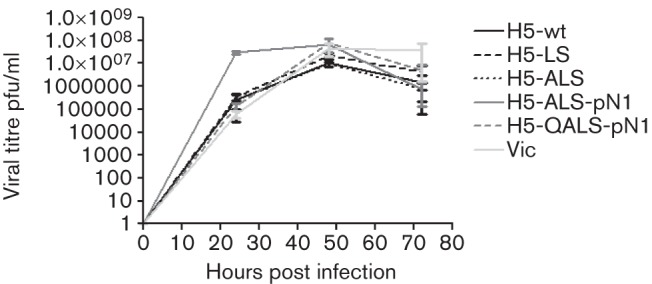
Growth of H5 HA mutant viruses in comparison to H5-wt and a recombinant virus bearing the surface antigens of a human H3N2 virus (VIC) under multicycle conditions in MDCK cells. Infectivity released into cell supernatant at various time points was titrated by plaque assay on MDCK cells. Each virus was assayed in triplicate and the mean p.f.u. ml^−1^ plotted with sd error bars. Student’s *t*-test was performed comparing each virus titre to that for H5-wt virus at each time point; H5-ALS-pN1 was significantly different at 24 h p.i. (***P<0.005). This result is representative of two independent experiments.

We next measured the multicycle replication of the recombinant viruses in MDCK cells. In this system all the recombinant viruses replicated robustly with released titres increasing between 24 and 48 h post-infection (p.i.). We did not observe any replicative advantage conferred by the H24Q pH stabilizing mutation in HA (Fig. 2).The combination of receptor binding changes in H5 HA with the NA gene from the pH 1N1 2009 virus (H5-ALS-pN1), did result in some enhancement of virus release at 24 h p.i. (*P*<0.005), likely due to an improvement in the balance between HA receptor binding and NA receptor destroying activity in MDCK cells. However, when the H24Q pH stabilizing mutation was added to this genetic constellation, the *in vitro* replicative advantage was tempered.

### A virus with a lower pH of fusion of HA replicates to higher titres in the ferret URT and transmits to a contact animal

We then ascertained that experiments using a PR8 internal gene constellation with human-adapted HA and NA genes could detect efficient infection and transmission in the ferret model. Following intranasal inoculation with 10^4^ p.f.u. of a recombinant virus with HA and NA gene segments of the human H3N2 virus (A/Victoria/3/75) on a PR8 internal genetic backbone, two donor animals were robustly infected. These animals shed infectious virus from the nose for the following 6 days, at titres in excess of 10^3^ p.f.u. ml^−1^ (Fig. S1, available in JGV Online). Moreover, the virus was transmitted to five out of six co-housed sentinel animals who themselves shed virus (Fig. S1).

We then assessed the replication of the H5 HA engineered viruses in the URT of ferrets and also tested whether the mutations affected the ability of the virus to pass from one ferret to another under direct contact conditions. These experiments were performed with the knowledge and approval of local and national safety committees including the Health and Safety Executive of the UK, under containment level 3 conditions, with ferrets housed in isolator cages and all persons involved in their care or in sampling procedures donning personal protective equipment including personal respirators.

**Fig. 3.**
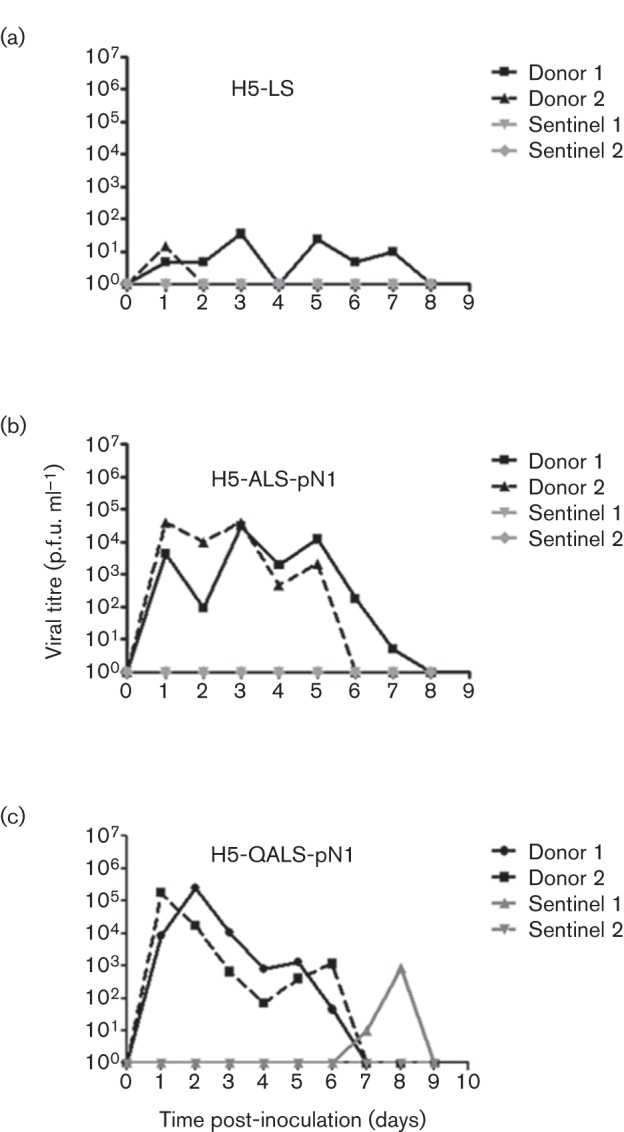
Two donor ferrets were inoculated intranasally with 10^6^ p.f.u. of each of the H5 HA mutant viruses (a) H5-LS, (b) H5-ALS-pN1 and (c) H5-QALS-pN1, then 24 h later each ferret was co-housed individually with a naive contact ferret (sentinel). All ferrets were nasal washed daily and viral titre determined by plaque assay on MDCK cells.

The peak titre of infectious virus shed by donor animals infected with 10^6^ p.f.u. of the recombinant virus H5-LS, which contained the Q226L and G228S mutations, was very low [<100 p.f.u. (ml nasal wash)^−1^] and no onwards transmission occurred even though the sentinel animals were co-housed (Fig. 3a, Table 2). Adding the T160A mutation and coupling this HA with the NA gene from a pH 1N1 virus (H5-ALS-pN1) resulted in inoculated ferrets shedding higher titres of virus, reaching 4×10^4^ p.f.u. (ml nasal wash)^−1^ at day 1–2 post-inoculation (Fig. 3b, Table 2). However, still no transmission to naive contact animals was observed by viral detection in daily nasal wash samples and neither of the contact animals showed H5 seroconversion 21 days after exposure (Table 2).

**Table 2.** Replication and contact transmission of H5 HA mutant viruses in ferrets Groups of two ferrets were inoculated with 10^6^ p.f.u. each virus and housed individually with a naive contact after 24 h. All ferrets were nasal washed daily and the viral titres determined by plaque assay on MDCK cells. Seroconversion was determined by microneutralization assay on homologous virus with sera collected at 21 days post exposure.

	Inoculated donor			Contact sentinel	
Virus name	Infected	Peak viral titre shed (p.f.u. ml^−1^)	Day of viral peak	Viral shedding	Seroconversion
H5-LS*	2/2	35	3	0/2	0/2
		15	1		
H5-ALS-pN1*	2/2	3×10^4^	3	0/2	0/2
		4×10^4^	3		
H5-QALS-pN1*	2/2	2.5×10^5^	2	1/2	1/2
		1.75×10^5^	1		

* Viruses contained the six internal gene segments from A/PR/8/34.

The introduction of the single point mutation H24Q resulted in robust infection of both donor animals with titres of shed virus achieving 2×10^5^ p.f.u. (ml nasal wash)^−1^ (Table 2). Moreover, there was virological and serological evidence of transmission to one of the two co-housed sentinel animals (Fig. 3c).

### Antibodies in sera from animals infected by the mutated H5 viruses cross-neutralize lentiviral pseudotypes with wild-type H5 HAs

Sera from animals infected with H5 viruses were tested for their ability to neutralize lentiviruses pseudotyped with H5 HA from either the clade 1 A/Vietnam/1194/2004 virus, VN04 (H5-wt), or the clade 2.2.1 A/turkey/Turkey/01/2005 virus (TKY05). The lentivirus pseudotype neutralization assay is a well-accepted and safe methodology for this purpose (Alberini *et al.*, 2009; Temperton *et al.*, 2007). As a positive control, a ferret serum was obtained from Diane Major, National Institute for Biological Standards and Control (NIBSC), South Mimms, UK, from ferrets infected with either wild-type A/Vietnam/1194/2004 virus (F1/04) or the attenuated vaccine virus RG14 which bears A/Vietnam/1194/2004 H5 HA with a modified HA cleavage site (F5/09). These sera were effective for neutralization of the homologous and heterologous H5-pseudotyped viruses (Table 3). In contrast, serum from a negative control ferret that was exposed but uninfected by pH 1N1 2009 virus (F100) had no neutralization activity against either H5 pseudotype (Table 3). Sera from animals infected with any of the H5 viruses with humanizing receptor mutations, including LS, ALS and QALS, were all able to neutralize entry of pseudotyped viruses with either of the wild-type avian H5 HAs. These observations show that the increased replication in the nose by viruses with H5 HA that is more pH stable than wild-type H5 virus led to the generation of robust titres of antibodies that were able to efficiently neutralize wild-type H5 viruses of a different clade.

**Table 3.** Neutralization of lentiviral pseudotypes by ferret sera

Ferret*	Description of serum	Titre vs A/Vietnam/1194/04 HA pseudotype	Titre vs A/turkey/Turkey/05 HA pseudotype
F1/04	p.i. wt A/Vietnam/1194/04	2383	2988
F5/09	p.i. RG14	604	798
F108	Donor p.i. LS	1791	524
F113	Donor p.i. ALS	4163	1606
F114	Donor p.i. ALS	<40	103
F182	Donor p.i. QALS	2083	1744
F183	Donor p.i. QALS	1137	3183
F180	Sentinel post-exposure QALS	702	452
F100	Donor post-exposure to pH 1N1 2009	<40	<40

* F1/04 and F5/09 ferret sera obtained from NIBSC.

## Discussion

Recently two laboratories showed that ferret-passaged highly pathogenic H5N1 influenza virus acquired aerosol transmissibility in ferrets following mutations in the HA gene that both enhanced the HA affinity for α2,6 linked SA and affected the stability of the HA molecule (Herfst *et al.*, 2012; Imai *et al.*, 2012). The viruses utilized for those two studies retained genetic characteristics associated with high virulence, for example, the multi-basic cleavage site of H5 HA and, in the case of the work from Herfst and colleagues, an internal genetic constellation derived from the H5N1 virus that may be associated with induction of a cytokine storm (Mok *et al.*, 2009). The work we describe here shares some similarities in approach with those two studies in that we have engineered humanizing mutations into influenza viruses of the H5 subtype. However, we chose to perform this work in a virus genetic background where the HA pathogenicity determinant (multi-basic cleavage site) was removed and the internal genes were replaced by those of the PR8 high growth reassortant vaccine donor strain, which has a long history of safe use. This undoubtedly led to a decrease in the levels of virus titres accumulated in the nose of infected ferrets compared with the wild-type genetic background. Indeed, a comparison of nasal virus titres shed from ferrets infected with the VIC/PR8 virus in Fig. S1 with titres on equivalent days shed from ferrets infected with a wild-type Victoria virus (Roberts *et al.*, 2011) indicated that the internal genes of PR8 reduced virus shedding by 2–3 logs. Thus, we do not suggest that we would measure respiratory droplet transmission using this genetic backbone, which is a stringent measure of mammalian adaptation. Nonetheless, our data suggest similar conclusions to those from the work with HPAI virus, that increased replication and consequent onwards transmission of H5 influenza virus may be increased in ferrets by combinations of mutations that affect at least three biological traits: alteration of receptor binding preference, stabilization of HA and a replication-competent polymerase (Imai *et al.*, 2012). In addition, our results suggest that a NA gene adapted to human transmission may enhance URT shedding and onwards transmissibility (Fig. 2). We did not formally establish the effect of the NA gene in this study, by comparing infection and transmissibility of, for example, an H5-ALS mutant possessing the Vietnam N1 NA with that of a virus containing the same HA coupled with pH 1N1 NA. However, several other studies have suggested a role for NA in transmission (Chen *et al.*, 2012a; Lakdawala & Subbarao, 2012; Lakdawala *et al.*, 2011) and our own studies had reinforced this (D. Blumenkrantz & W. S. Barclay, unpublished data). Therefore, we chose to use the pH 1N1 NA in the present studies in order to allow us to focus on the effects of changes in the H5 HA.

Of these proposed adaptations, the requirement for pH stabilization is the most novel. The pH at which HA fusion occurs is a compromise between the need for virus to survive in the environment and the enhancement to infectivity that can be conferred by fusion and uncoating in early rather than in late endosomes. This balance will be altered when the virus crosses from one species to another and finds a new environmental niche across which transmission must occur. The fusion pH of HA has already been shown to be important in the transmission of H5N1 viruses in ducks. In this natural avian host, mutations that stabilized HA, decreasing the pH of fusion below pH 5.6, resulted in a reduction in bird to bird transmission (Reed *et al.*, 2010). It was also recently shown that the pathogenicity of HPAI H5N1 in chickens is affected by the pH of HA fusion, with an increased pH of fusion leading to a more pathogenic phenotype *in vivo* (DuBois *et al.*, 2011). Thus, it seems that in birds, the advantages conferred by rapid entry and uncoating outweigh the need for pH stability during the transmission event. However, we found that influenza viruses that are transmissible in humans, of both influenza type A and B, did not undergo HA fusion until the pH was less than 5.3 (Fig. 1a). The human nasal cavity has a pH ranging from 5.1–8.1, suggesting the environment from which influenza virus is shed and into which it must survive during transmission to a new host can be moderately acidic (England *et al.*, 1999; Washington *et al.*, 2000). It has also been shown that the NA with which any given influenza HA is coupled can also influence the pH at which a virus mediates the fusion event, probably by affecting the access of HA for the SA receptor (DuBois *et al.*, 2011). In our study, we did not find an effect of NA on the pH of HA fusion, rather the HA mutation in the stalk region of HA1 subunit had the dominant effect.

The mutation we used to stabilize H5 HA, H24Q, was introduced into the H5 HA of A/Vietnam/1194/2004, a clade 1 virus for which a licensed pre-pandemic vaccine, (Prepandrix; GlaxoSmithKline Biologicals) has been generated and for which a crystal structure is available (Stevens *et al.*, 2006; Yamada *et al.*, 2006). This residue is located in the fusion peptide pocket of the H5 HA, where it is one of three conserved histidines that at low pH become positively charged and cause the fusion peptide to exit the pocket in an irreversible conformational change (Stevens *et al.*, 2004). Reed *et al.* (2009) already demonstrated that the H24Q mutation reduced the pH of fusion of a closely related H5N1 virus (A/chicken/Vietnam/C58/2004). However, there are several other genetic changes by which the H5 HA might acquire increased pH stability. Interestingly, during the independent studies involving ferret passage leading to transmission of recombinant H5N1 influenza viruses, different mutations in the HA stalk region were selected that resulted in increased pH stability (Herfst *et al.*, 2012; Imai *et al.*, 2012). Mutations that H5N1 viruses are accumulating whilst circulating in the wild bird population are currently monitored for changes to the receptor binding site of the HA protein (Watanabe *et al.*, 2011). It now seems important to also monitor for changes that affect pH stability so that viruses with increased pandemic potential that arise in nature can be detected early.

Ideally, during this pre-pandemic grace period we will improve our ability to generate effective vaccines against novel influenza subtypes. LAIVs for seasonal human influenza are used widely in some countries, especially in children, an important target group who are major spreaders of virus through the community (Carrat *et al.*, 2006; Cauchemez *et al.*, 2011; Loeb *et al.*, 2010). However, pre-pandemic avian influenza LAIVs currently induce only a poor immune response due to the conflict between their restriction to the URT by cold-adapted internal genes and the lack of α2,6 SA tropism of their HA. Moves to increase the ‘take rate’ of LAIVs by altering receptor binding or lowering the pH of fusion have both been explored separately (Krenn *et al.*, 2011; Wang *et al*., 2010). Here, we combined these two approaches and showed an additive effect. It may be useful to use one or other strategy separately to generate an H5 HA LAIV with higher nasal replication but which is still unable to transmit onwards to non-immunized individuals. Most important though, is the assessment of whether by engineering mutations into the H5 HA that increase nasal replication there is any unforeseen effect on antigenicity that might compromise the efficiency of the induced immune response to protect vaccinees. Wang *et al.* (2010) made H5N1 LAIV with humanizing mutations in the HA receptor binding site, including deletion of the glycosylation site at residue 158, and found that the sera generated in ferrets infected with these vaccine candidates were inefficient at recognizing the wild-type H5 HA (Wang *et al.*, 2010). This may be because glycosylation of T158 interferes with antibody access to an important epitope (Bender *et al.*, 1999). Similarly Chen *et al.* (2012b), striving to increase the vaccine immunogenicity of LAIV against H2 and H6 avian subtypes of influenza by engineering humanizing mutations in the receptor binding site, also found that although homologous titres were increased, the sera induced by immunization with the viruses with mutated HA did not cross-protect efficiently against challenge with virus that retained wild-type avian HA. In our study we demonstrated using ferret sera from animals infected with humanized H5 HA viruses that both receptor switching mutations and pH stability alterations led to a robust immune response, and the resulting sera could neutralize lentivirus pseudotyped with homologous wild-type H5 HA (Table 3). Moreover, in animals infected with the ‘double’ QALS mutant, a considerable titre of cross-clade neutralizing antibody titre was induced without compromising the titre against the homologous wild-type H5 HA. This suggests that even when wild-type HA retains glycosylation that may mask some antigenic epitopes, the cost to neutralizing ability can be compensated for by inducing a stronger immune response.

Since the exact nature of mutations that will ultimately arise in nature and lead to transmissible H5 virus cannot be exactly predicted, it will be important to continue research aimed to understand how mutations in receptor binding and pH stability affect the immune response generated by recombinant virus vaccines. Overall, this work has important implications for the rational design of LAIVs or more traditional pre-pandemic H5N1 vaccines, as well as for understanding the barriers that limit avian influenza virus transmissibility in mammals.

## Methods

### 

#### Reagents, cells and viruses.

293T and MDCK cells were maintained in Dulbecco’s modified 269 Eagle’s medium (DMEM; Gibco, Invitrogen) supplemented with 10 % FBS (Biosera) and 1% penicillin/streptomycin (Gibco, Invitrogen) in 5% CO_2_. Wild-type human viruses, A/Sydney/2007/97 (H3N2), A/Victoria/3/75 (H3N2), A/England/195/09 pH 1N1 and B/Florida/04/06, and recombinant viruses as below were grown in MDCK cells.

#### Generation of H5 HA mutant viruses.

Recombinant viruses were generated by 12 plasmid RG in 293T and MDCK cells as described elsewhere (Elleman & Barclay, 2004; Neumann & Kawaoka, 1999). Recombinant viruses possessed the six internal genes (PB1, PB2, PA, NP, M and NS) from the high growth reassortant vaccine donor strain A/PR/8/34 and the wild-type or mutated surface proteins HA and NA from the following strains: A/Vietnam/1194/04 (H5N1), A/turkey/Turkey/01/05 (H5N1), A/Hong Kong/156/97 (H5N1) or A/chicken/Italy/13474/99 (H7N1). All H5N1 and H7N1 viruses were generated from plasmids where the multi-basic site was replaced with a mono-basic cleavage site known to ablate the high pathogenicity of these HAs. Site-directed mutagenesis on the H5 HA gene from A/Vietnam/1194/04 generated receptor binding mutations (T160A, Q226L and G228S) and the pH stabilizing mutation (H24Q) in various combinations (see Table 1 for virus genetic details).

#### pH of fusion assays.

Assays of the pH for HA fusion were carried out as described by Krenn *et al.* (2011) with the following modifications: 64 HA units per 50 µl each virus were mixed with 150 µl 1 % (w/v) suspension of hRBCs in PBS and incubated at 4 °C for 1 h to allow HA attachment to SA. The virus and hRBC mix was then pelleted, washed in PBS and resuspended in MES buffer of varying pH values (from pH 4.6 to 6.0) followed by incubation at 37 °C for 60 min to allow fusion. Fusion results in release of haemoglobin from the hRBCs, which can be quantified by measuring the optical density at 405 nm. A mock control, without any virus, was performed for each pH value to control for effects of lowered pH on the stability of the hRBCs. The mock value for optical density at 405 nm for each pH value was subtracted from each virus reading, and then the percentage haemolysis was expressed as a percentage of the maximal reading for each virus. At the lowest pH of 4.6, this background level of haemolysis was not more than 20 % of the raw optical density value obtained with any virus. Every virus at each pH condition was performed in quadruplet and plotted as a mean with sd as error bars.

#### MDCK growth curves.

Confluent MDCK cells in triplicate were infected with an m.o.i. of 0.001 of each virus for 1 h at 37 °C before the inocula were removed and replaced with serum free DMEM (Gibco, Invitrogen) plus 1.4 µg TPCK trypsin ml^−1^ (Worthington). Virus used for inoculation was back-titrated on the day (time 0) by plaque assay on MDCK cells to confirm that inputs contained equal m.o.i. Cells were incubated at 32 °C and at 24, 48 and 72 h p.i. an aliquot of the medium was taken from each well and titrated via plaque assay on MDCK cells. Mean p.f.u. ml^−1^ was plotted with sd as error bars. An unpaired *t*-test was used to assess the statistical significance between viruses.

#### Ferret studies.

All experimental conditions were approved by local and national safety committees including the Health and Safety Executive in the UK. The experimental set up was approved also by the Home Office in the UK, who licence experimental use of animals. Female ferrets (14–16 weeks old) weighing 700–900 g were used. After acclimatization, sera were obtained and tested by micro-neutralization assay for antibodies against A/England/195/09, a prototypic 2009 H1N1 influenza virus, which was the predominant virus circulating in the community at the time of these experiments. All ferrets were negative for influenza antibodies at the start of the experiments. All ferret studies were performed in a containment level 3 laboratory with the ferrets housed in HEPA (high efficiency particulate air)-filtered isolators then nasal washed and weighed over a downdraft table. Ferrets were moved between the isolator and downdraft table in a HEPA-filtered transport box so the ferrets were under primary containment at all times except whilst on the downdraft table. All personnel involved in the care and experimental sampling of the ferrets wore personal protective equipment, including personal 3M filtered respirators. Ferret body weight was measured daily. Sentinel animals were handled before inoculated animals, and work surfaces decontaminated and handlers’ gloves exchanged between animals. For inoculation, ferrets were lightly anaesthetized with ketamine (22 mg kg^−1^) and xylazine (0.9 mg kg^−1^), and inoculated intranasally with 10^6^ p.f.u. virus diluted in PBS (0.1 ml per nostril) on the downdraft table. Each of two inoculated donor ferrets was co-housed after 24 h with one naive sentinel animal. All animals were nasal washed daily, while conscious, by instilling 2 ml PBS into the nostrils, and the expectorate was collected in modified 250 ml centrifuge tubes. BSA (0.3 %) was added to the nasal wash expectorate before infectious virus was titrated by plaque assay on MDCK cells. The limit of virus detection in the plaque assays was 10 p.f.u. ml^−1^. Ferrets were housed for 21 days from inoculation when they were humanely euthanized and terminal blood collected for analysis by microneutralization assay.

#### Pseudotype construction and neutralization assays.

Lentiviral pseudotypes with HA glycoproteins derived from the HPAI viruses H5N1 clade 1 A/Vietnam/1194/04 and clade 2 A/turkey/Turkey/01/05 were constructed as described previously (Temperton *et al.*, 2007). Pseudotype viruses were produced by co-transfection of 293T cells with the respective HA plasmids, the human immunodeficiency virus gag-pol plasmid p8.91 and the reporter plasmid pCSFLW (expressing firefly luciferase) using the FuGENE-6 transfection reagent (Roche). For the neutralization assay, serum samples (5 µl) were heat inactivated at 56 °C for 30 min, twofold serially diluted in culture medium and mixed with pseudotype virus [1×10^6^ relative light units (RLU) luciferase input] at a 1 : 1 (v/v) ratio. After incubation at 37 °C for 1 h, 1×10^4^ 293T cells were added to each well of a white 96-well flat-bottomed tissue culture plate. Firefly RLU were evaluated 48 h later by luminometry using the Bright-Glo assay system (Promega). IC_50_ end-point titres were calculated using GraphPad Prism software from RLU values obtained from duplicate serum assays and from pseudotype virus only/cell only controls.

## References

[r1] Aditama T. Y. Samaan G. Kusriastuti R. Sampurno O. D. Purba W. Misriyah Santoso H. Bratasena A. Maruf A. **& other authors (**2012**).** Avian influenza H5N1 transmission in households, Indonesia. PLoS ONE 7, e29971 10.1371/journal.pone.0029971 22238686 PMC3251608

[r2] Alberini I. Del Tordello E. Fasolo A. Temperton N. J. Galli G. Gentile C. Montomoli E. Hilbert A. K. Banzhoff A. **& other authors (**2009**).** Pseudoparticle neutralization is a reliable assay to measure immunity and cross-reactivity to H5N1 influenza viruses. Vaccine 27, 5998–6003 10.1016/j.vaccine.2009.07.079 19665606

[r3] Auewarakul P. Suptawiwat O. Kongchanagul A. Sangma C. Suzuki Y. Ungchusak K. Louisirirotchanakul S. Lerdsamran H. Pooruk P. **& other authors (**2007**).** An avian influenza H5N1 virus that binds to a human-type receptor. J Virol 81, 9950–9955 10.1128/JVI.00468-07 17626098 PMC2045398

[r4] Ayora-Talavera G. Shelton H. Scull M. A. Ren J. Jones I. M. Pickles R. J. Barclay W. S. **(**2009**).** Mutations in H5N1 influenza virus hemagglutinin that confer binding to human tracheal airway epithelium. PLoS ONE 4, e7836 10.1371/journal.pone.0007836 19924306 PMC2775162

[r5] Bender C. Hall H. Huang J. Klimov A. Cox N. Hay A. Gregory V. Cameron K. Lim W. Subbarao K. **(**1999**).** Characterization of the surface proteins of influenza A (H5N1) viruses isolated from humans in 1997-1998. Virology 254, 115–123 10.1006/viro.1998.9529 9927579

[r6] Beyer W. E. P. Ruigrok R. W. H. van Driel H. Masurel N. **(**1986**).** Influenza virus strains with a fusion threshold of pH 5.5 or lower are inhibited by amantadine. Arch Virol 90, 173–181 10.1007/BF01314156 3089198

[r7] Carrat F. Luong J. Lao H. Sallé A. V. Lajaunie C. Wackernagel H. **(**2006**).** A ‘small-world-like’ model for comparing interventions aimed at preventing and controlling influenza pandemics. BMC Med 4, 26 10.1186/1741-7015-4-26 17059593 PMC1626479

[r8] Cauchemez S. Bhattarai A. Marchbanks T. L. Fagan R. P. Ostroff S. Ferguson N. M. Swerdlow D. Sodha S. V. Moll M. E. **& other authors (**2011**).** Role of social networks in shaping disease transmission during a community outbreak of 2009 H1N1 pandemic influenza. Proc Natl Acad Sci U S A 108, 2825–2830 10.1073/pnas.1008895108 21282645 PMC3041067

[r9] Chen L. M. Blixt O. Stevens J. Lipatov A. S. Davis C. T. Collins B. E. Cox N. J. Paulson J. C. Donis R. O. **(**2012a**).** *In vitro* evolution of H5N1 avian influenza virus toward human-type receptor specificity. Virology 422, 105–113 10.1016/j.virol.2011.10.006 22056389 PMC5480292

[r10] Chen Z. Zhou H. Kim L. Jin H. **(**2012b**).** The receptor binding specificity of the live attenuated influenza H2 and H6 vaccine viruses contributes to vaccine immunogenicity and protection in ferrets. J Virol 86, 2780–2786 10.1128/JVI.06219-11 22190726 PMC3302243

[r11] Chutinimitkul S. van Riel D. Munster V. J. van den Brand J. M. Rimmelzwaan G. F. Kuiken T. Osterhaus A. D. Fouchier R. A. de Wit E. **(**2010**).** *In vitro* assessment of attachment pattern and replication efficiency of H5N1 influenza A viruses with altered receptor specificity. J Virol 84, 6825–6833 10.1128/JVI.02737-09 20392847 PMC2903244

[r12] Couch R. B. Keitel W. A. Cate T. R. **(**1997**).** Improvement of inactivated influenza virus vaccines. J Infect Dis 176 (Suppl. 1), S38–S44 10.1086/514173 9240693

[r13] DuBois R. M. Zaraket H. Reddivari M. Heath R. J. White S. W. Russell C. J. **(**2011**).** Acid stability of the hemagglutinin protein regulates H5N1 influenza virus pathogenicity. PLoS Pathog 7, e1002398 10.1371/journal.ppat.1002398 22144894 PMC3228800

[r14] Elleman C. J. Barclay W. S. **(**2004**).** The M1 matrix protein controls the filamentous phenotype of influenza A virus. Virology 321, 144–153 10.1016/j.virol.2003.12.009 15033573

[r15] England R. J. Homer J. J. Knight L. C. Ell S. R. **(**1999**).** Nasal pH measurement: a reliable and repeatable parameter. Clin Otolaryngol Allied Sci 24, 67–68 10.1046/j.1365-2273.1999.00223.x 10196653

[r16] Gambaryan A. Tuzikov A. Pazynina G. Bovin N. Balish A. Klimov A. **(**2006**).** Evolution of the receptor binding phenotype of influenza A (H5) viruses. Virology 344, 432–438 10.1016/j.virol.2005.08.035 16226289

[r17] Gao Y. Zhang Y. Shinya K. Deng G. Jiang Y. Li Z. Guan Y. Tian G. Li Y. **& other authors (**2009**).** Identification of amino acids in HA and PB2 critical for the transmission of H5N1 avian influenza viruses in a mammalian host. PLoS Pathog 5, e1000709 10.1371/journal.ppat.1000709 20041223 PMC2791199

[r18] Giannecchini S. Campitelli L. Calzoletti L. De Marco M. A. Azzi A. Donatelli I. **(**2006**).** Comparison of *in vitro* replication features of H7N3 influenza viruses from wild ducks and turkeys: potential implications for interspecies transmission. J Gen Virol 87, 171–175 10.1099/vir.0.81187-0 16361429

[r19] Harvey R. Martin A. C. Zambon M. Barclay W. S. **(**2004**).** Restrictions to the adaptation of influenza A virus H5 hemagglutinin to the human host. J Virol 78, 502–507 10.1128/JVI.78.1.502-507.2004 14671130 PMC303389

[r20] Hatta M. Gao P. Halfmann P. Kawaoka Y. **(**2001**).** Molecular basis for high virulence of Hong Kong H5N1 influenza A viruses. Science 293, 1840–1842 10.1126/science.1062882 11546875

[r21] Herfst S. Schrauwen E. J. Linster M. Chutinimitkul S. de Wit E. Munster V. J. Sorrell E. M. Bestebroer T. M. Burke D. F. **& other authors (**2012**).** Airborne transmission of influenza A/H5N1 virus between ferrets. Science 336, 1534–1541 10.1126/science.1213362 22723413 PMC4810786

[r22] Horimoto T. Kawaoka Y. **(**2009**).** Designing vaccines for pandemic influenza. Curr Top Microbiol Immunol 333, 165–176 10.1007/978-3-540-92165-3_8 19768405 PMC6133292

[r23] Imai M. Watanabe T. Hatta M. Das S. C. Ozawa M. Shinya K. Zhong G. Hanson A. Katsura H. **& other authors (**2012**).** Experimental adaptation of an influenza H5 HA confers respiratory droplet transmission to a reassortant H5 HA/H1N1 virus in ferrets. Nature 486, 420–428 22722205 10.1038/nature10831 PMC3388103

[r24] Karron R. A. Talaat K. Luke C. Callahan K. Thumar B. Dilorenzo S. McAuliffe J. Schappell E. Suguitan A. **& other authors (**2009**).** Evaluation of two live attenuated cold-adapted H5N1 influenza virus vaccines in healthy adults. Vaccine 27, 4953–4960 10.1016/j.vaccine.2009.05.099 19540952 PMC4806665

[r25] Krenn B. M. Egorov A. Romanovskaya-Romanko E. Wolschek M. Nakowitsch S. Ruthsatz T. Kiefmann B. Morokutti A. Humer J. **& other authors (**2011**).** Single HA2 mutation increases the infectivity and immunogenicity of a live attenuated H5N1 intranasal influenza vaccine candidate lacking NS1. PLoS ONE 6, e18577 10.1371/journal.pone.0018577 21490925 PMC3072404

[r26] Lakdawala S. S. Subbarao K. **(**2012**).** The ongoing battle against influenza: the challenge of flu transmission. Nat Med 18, 1468–1470 10.1038/nm.2953 23042349

[r27] Lakdawala S. S. Lamirande E. W. Suguitan A. L. Jr Wang W. Santos C. P. Vogel L. Matsuoka Y. Lindsley W. G. Jin H. Subbarao K. **(**2011**).** Eurasian-origin gene segments contribute to the transmissibility, aerosol release, and morphology of the 2009 pandemic H1N1 influenza virus. PLoS Pathog 7, e1002443 10.1371/journal.ppat.1002443 22241979 PMC3248560

[r28] Lin Y. P. Wharton S. A. Martín J. Skehel J. J. Wiley D. C. Steinhauer D. A. **(**1997**).** Adaptation of egg-grown and transfectant influenza viruses for growth in mammalian cells: selection of hemagglutinin mutants with elevated pH of membrane fusion. Virology 233, 402–410 10.1006/viro.1997.8626 9217063

[r29] Loeb M. Russell M. L. Moss L. Fonseca K. Fox J. Earn D. J. Aoki F. Horsman G. Van Caeseele P. **& other authors (**2010**).** Effect of influenza vaccination of children on infection rates in Hutterite communities: a randomized trial. JAMA *(*J Am Med Assoc*)* 303, 943–950 10.1001/jama.2010.250 20215608

[r30] Maines T. R. Chen L. M. Van Hoeven N. Tumpey T. M. Blixt O. Belser J. A. Gustin K. M. Pearce M. B. Pappas C. **& other authors (**2011**).** Effect of receptor binding domain mutations on receptor binding and transmissibility of avian influenza H5N1 viruses. Virology 413, 139–147 10.1016/j.virol.2011.02.015 21397290 PMC5470842

[r31] Mok K. P. Wong C. H. Cheung C. Y. Chan M. C. Lee S. M. Nicholls J. M. Guan Y. Peiris J. S. **(**2009**).** Viral genetic determinants of H5N1 influenza viruses that contribute to cytokine dysregulation. J Infect Dis 200, 1104–1112 10.1086/605606 19694514 PMC4028720

[r32] Neumann G. Kawaoka Y. **(**1999**).** Genetic engineering of influenza and other negative-strand RNA viruses containing segmented genomes. Adv Virus Res 53, 265–300 10.1016/S0065-3527(08)60352-8 10582103

[r33] Reed M. L. Yen H. L. DuBois R. M. Bridges O. A. Salomon R. Webster R. G. Russell C. J. **(**2009**).** Amino acid residues in the fusion peptide pocket regulate the pH of activation of the H5N1 influenza virus hemagglutinin protein. J Virol 83, 3568–3580 10.1128/JVI.02238-08 19193808 PMC2663236

[r34] Reed M. L. Bridges O. A. Seiler P. Kim J. K. Yen H. L. Salomon R. Govorkova E. A. Webster R. G. Russell C. J. **(**2010**).** The pH of activation of the hemagglutinin protein regulates H5N1 influenza virus pathogenicity and transmissibility in ducks. J Virol 84, 1527–1535 10.1128/JVI.02069-09 19923184 PMC2812356

[r35] Roberts K. L. Shelton H. Scull M. Pickles R. Barclay W. S. **(**2011**).** Lack of transmission of a human influenza virus with avian receptor specificity between ferrets is not due to decreased virus shedding but rather a lower infectivity *in vivo*. J Gen Virol 92, 1822–1831 10.1099/vir.0.031203-0 21508186

[r36] Skehel J. J. Wiley D. C. **(**2000**).** Receptor binding and membrane fusion in virus entry: the influenza hemagglutinin. Annu Rev Biochem 69, 531–569 10.1146/annurev.biochem.69.1.531 10966468

[r37] Stevens J. Corper A. L. Basler C. F. Taubenberger J. K. Palese P. Wilson I. A. **(**2004**).** Structure of the uncleaved human H1 hemagglutinin from the extinct 1918 influenza virus. Science 303, 1866–1870 10.1126/science.1093373 14764887

[r38] Stevens J. Blixt O. Tumpey T. M. Taubenberger J. K. Paulson J. C. Wilson I. A. **(**2006**).** Structure and receptor specificity of the hemagglutinin from an H5N1 influenza virus. Science 312, 404–410 10.1126/science.1124513 16543414

[r39] Su Y. Yang H. Y. Zhang B. J. Jia H. L. Tien P. **(**2008**).** Analysis of a point mutation in H5N1 avian influenza virus hemagglutinin in relation to virus entry into live mammalian cells. Arch Virol 153, 2253–2261 10.1007/s00705-008-0255-y 19020946

[r40] Subbarao K. Murphy B. R. Fauci A. S. **(**2006**).** Development of effective vaccines against pandemic influenza. Immunity 24, 5–9 10.1016/j.immuni.2005.12.005 16413916

[r41] Temperton N. J. Hoschler K. Major D. Nicolson C. Manvell R. Hien V. M. Ha D. Q. de Jong M. Zambon M. **& other authors (**2007**).** A sensitive retroviral pseudotype assay for influenza H5N1-neutralizing antibodies. Influenza Other Respi Viruses 1, 105–112 10.1111/j.1750-2659.2007.00016.x 19453415 PMC4941878

[r42] Ungchusak K. Auewarakul P. Dowell S. F. Kitphati R. Auwanit W. Puthavathana P. Uiprasertkul M. Boonnak K. Pittayawonganon C. **& other authors (**2005**).** Probable person-to-person transmission of avian influenza A (H5N1). N Engl J Med 352, 333–340 10.1056/NEJMoa044021 15668219

[r43] Wang H. Feng Z. Shu Y. Yu H. Zhou L. Zu R. Huai Y. Dong J. Bao C. **& other authors (**2008**).** Probable limited person-to-person transmission of highly pathogenic avian influenza A (H5N1) virus in China. Lancet 371, 1427–1434 10.1016/S0140-6736(08)60493-6 18400288

[r44] Wang W. Lu B. Zhou H. Suguitan A. L. Jr Cheng X. Subbarao K. Kemble G. Jin H. **(**2010**).** Glycosylation at 158N of the hemagglutinin protein and receptor binding specificity synergistically affect the antigenicity and immunogenicity of a live attenuated H5N1 A/Vietnam/1203/2004 vaccine virus in ferrets. J Virol 84, 6570–6577 10.1128/JVI.00221-10 20427525 PMC2903256

[r45] Washington N. Steele R. J. Jackson S. J. Bush D. Mason J. Gill D. A. Pitt K. Rawlins D. A. **(**2000**).** Determination of baseline human nasal pH and the effect of intranasally administered buffers. Int J Pharm 198, 139–146 10.1016/S0378-5173(99)00442-1 10767563

[r46] Watanabe Y. Ibrahim M. S. Ellakany H. F. Kawashita N. Mizuike R. Hiramatsu H. Sriwilaijaroen N. Takagi T. Suzuki Y. Ikuta K. **(**2011**).** Acquisition of human-type receptor binding specificity by new H5N1 influenza virus sublineages during their emergence in birds in Egypt. PLoS Pathog 7, e1002068 10.1371/journal.ppat.1002068 21637809 PMC3102706

[r47] WHO (2012a**).** *Antigenic and Genetic Characteristics of Zoonotic Influenza Viruses and the Development of Candidate Vaccines for Pandemic Prepardness* Geneva: World Health Organization.

[r48] WHO (2012b**).** *Cumulative Number of Confirmed Human Cases for Avian Influenza A (H5N1) Reported to WHO, 2003–2012* Geneva: World Health Organization.

[r49] Yamada S. Suzuki Y. Suzuki T. Le M. Q. Nidom C. A. Sakai-Tagawa Y. Muramoto Y. Ito M. Kiso M. **& other authors (**2006**).** Haemagglutinin mutations responsible for the binding of H5N1 influenza A viruses to human-type receptors. Nature 444, 378–382 10.1038/nature05264 17108965

